# Supplementary material for: Concentrations of Plasma Amino Acids and Neurotransmitters in Participants with Functional Gut Disorders and Healthy Controls
Source: Metabolites. 2023 Feb 20;13(2):313. doi: 10.3390/metabo13020313 (PMC9959678; doi:10.3390/metabo13020313)
Supplement: Supplementary file 1 [file metabolites-13-00313-s001.zip › metabolites-2142707-supplementary.pdf]

Supplementary Figures:

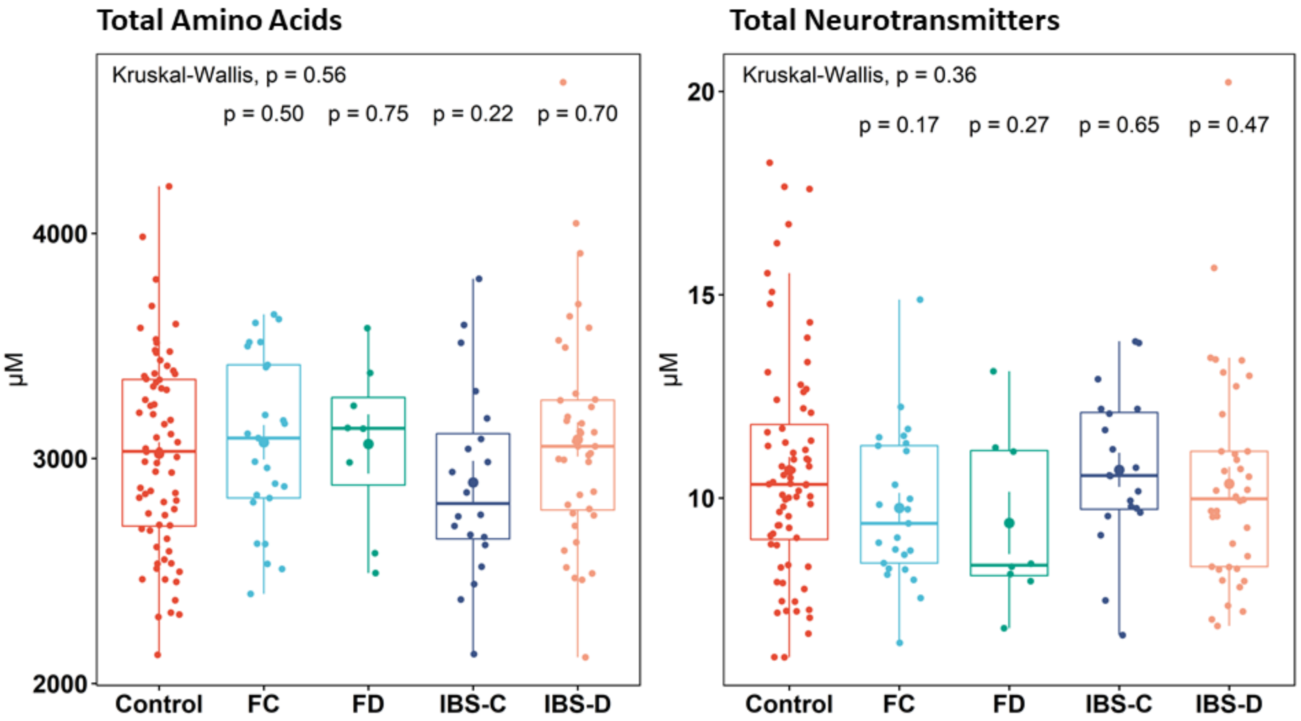

Supplementary Figure 1: Total sum of all amino acids (including non-proteogenic) and all neurotransmitters between healthy control, IBS subtypes, and functional groups. Top significance value denotes the overall significance between all groups. Individual group significance values are pairwise comparisons between healthy controls and the group. Boxplots show median (centre line), 25th and 75th percentile (top and bottom of boxes, respectively), with whiskers representing 1.5 times the inter-quartile range. Abbreviations: healthy control (control), functional constipation (FC), IBS-constipation (IBS-C), functional diarrhea (FD), IBS-diarrhea (IBS-D).

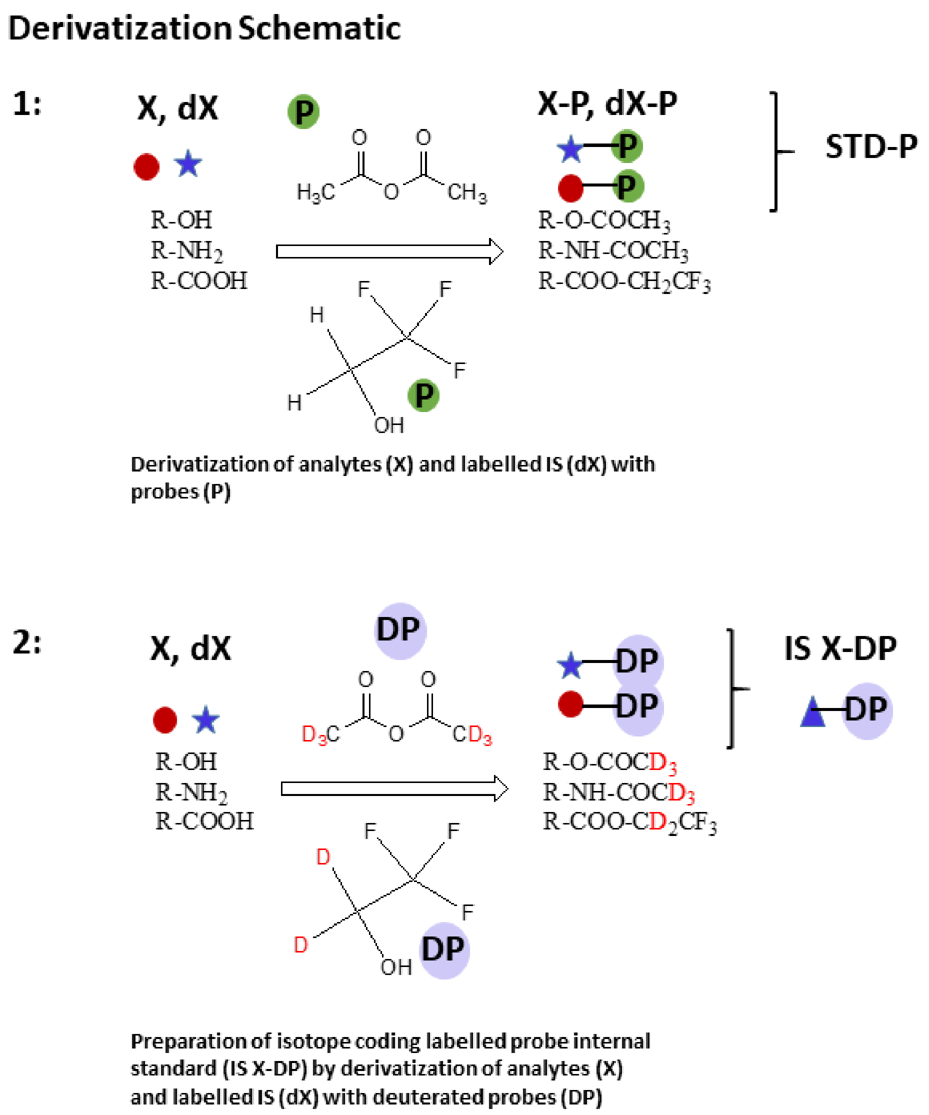

Supplementary Figure 2: Derivatization schematic for plasma neurotransmitter analysis.

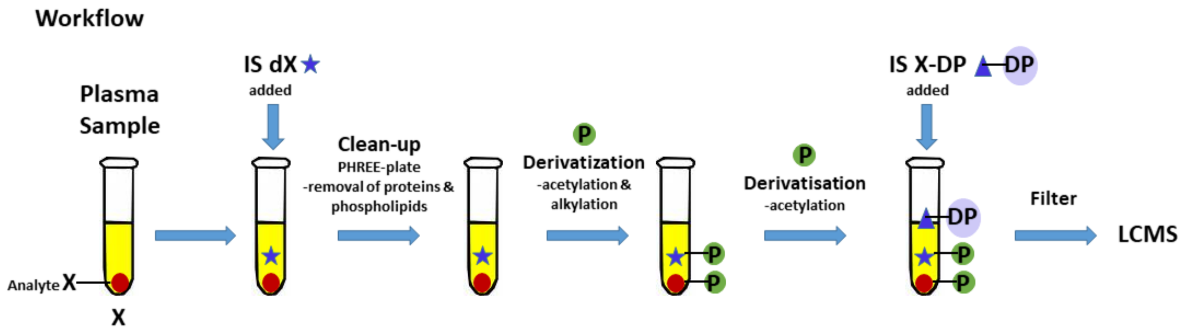

Supplementary Figure 3: Derivatization workflow for plasma neurotransmitter analysis.

Supplementary Table 1: Output of spearman's rank sum correlation test for healthy control group. Values shown as Rho/significance value.

	PEA	DOPA	DA	X3MT	DOPAC	HVA	NE	DHPG	MHPG	NM	E	MN	VMA	KYN	5.HTP	5.HT	5.HIAA	AABA	GABA
**ASP**	-0.26/0.03	-0.14/0.25	0.19/0.11	0.06/0.60	-0.20/0.10	0.22/0.07	0.02/0.85	0.06/0.62	0.06/0.62	0.05/0.70	-0.16/0.17	0.24/0.04	0.21/0.08	-0.20/0.09	-0.10/0.38	0.29/0.01	0.20/0.09	0.06/0.62	0.31/0.01
**GLU**	-0.20/0.10	-0.12/0.30	0.28/0.02	0.13/0.26	-0.08/0.49	0.18/0.12	-0.11/0.37	-0.19/0.12	-0.10/0.38	0.13/0.27	-0.19/0.11	0.07/0.55	0.06/0.60	-0.05/0.65	-0.04/0.73	-0.07/0.57	0.23/0.06	0.15/0.20	0.27/0.02
**HyPro**	0.03/0.79	-0.07/0.54	0.14/0.24	0.17/0.16	0.10/0.41	0.28/0.02	-0.06/0.61	-0.18/0.12	-0.13/0.28	-0.10/0.41	-0.22/0.07	0.24/0.04	0.04/0.72	0.01/0.93	-0.08/0.49	-0.05/0.65	0.09/0.47	0.17/0.15	0.22/0.06
**SER**	-0.07/0.56	0.15/0.21	0.14/0.25	-0.01/0.93	0.01/0.93	0.05/0.69	0.31/0.01	0.34/0.00	0.13/0.28	0.01/0.91	0.22/0.07	0.14/0.24	0.05/0.65	0.00/0.97	0.19/0.10	0.29/0.02	0.04/0.72	-0.04/0.73	-0.16/0.18
**ASN**	-0.16/0.19	0.22/0.06	0.13/0.26	-0.03/0.81	0.15/0.22	0.13/0.26	0.34/0.00	0.36/0.00	0.08/0.48	-0.02/0.88	0.21/0.07	0.25/0.03	0.24/0.04	0.14/0.25	0.29/0.01	0.27/0.02	0.13/0.29	0.03/0.80	-0.04/0.75
**GLY**	-0.07/0.55	0.14/0.24	-0.01/0.94	0.07/0.53	0.03/0.83	-0.04/0.73	0.11/0.35	0.09/0.47	0.00/1.00	-0.29/0.01	0.11/0.36	-0.04/0.73	0.08/0.53	0.06/0.62	-0.07/0.58	0.01/0.93	0.08/0.53	0.01/0.92	-0.02/0.85
**GLN**	-0.03/0.80	0.15/0.21	0.07/0.58	-0.05/0.69	0.04/0.75	-0.08/0.49	0.28/0.02	0.14/0.24	0.04/0.73	-0.07/0.54	0.02/0.87	0.05/0.70	0.11/0.34	-0.03/0.78	0.13/0.28	0.09/0.46	0.12/0.30	0.03/0.82	-0.13/0.29
**HIS**	0.03/0.83	0.04/0.73	0.20/0.10	0.06/0.62	-0.07/0.56	-0.12/0.33	0.14/0.26	-0.02/0.90	0.02/0.89	0.04/0.75	-0.02/0.87	0.15/0.21	-0.13/0.29	-0.02/0.88	0.14/0.24	0.19/0.12	0.19/0.11	0.04/0.76	0.10/0.40
**TAU**	-0.15/0.21	0.08/0.52	0.10/0.41	0.01/0.96	-0.23/0.05	0.08/0.49	0.33/0.00	0.32/0.01	0.35/0.00	0.08/0.50	0.13/0.29	0.02/0.86	0.33/0.00	-0.15/0.20	-0.02/0.84	0.43/0.00	0.28/0.02	-0.06/0.61	0.22/0.07
**CIT**	0.10/0.40	-0.25/0.03	0.05/0.65	-0.08/0.52	-0.10/0.39	-0.09/0.43	-0.01/0.90	0.09/0.45	0.01/0.90	0.08/0.49	-0.01/0.91	0.12/0.30	0.22/0.06	-0.11/0.37	-0.17/0.15	-0.03/0.81	0.02/0.90	0.05/0.67	0.16/0.18
**THR**	0.01/0.92	0.08/0.52	0.06/0.60	-0.03/0.83	0.14/0.23	0.01/0.94	0.12/0.31	0.03/0.79	-0.10/0.41	-0.08/0.48	0.09/0.45	0.26/0.03	0.00/0.98	0.16/0.19	0.17/0.15	0.08/0.49	0.14/0.25	0.19/0.11	0.03/0.77
**ALA**	-0.11/0.34	-0.01/0.94	0.13/0.27	-0.08/0.53	-0.01/0.91	0.12/0.30	0.02/0.88	-0.05/0.66	-0.20/0.10	-0.03/0.83	-0.19/0.10	0.11/0.37	0.19/0.10	0.01/0.92	0.19/0.10	0.09/0.47	0.19/0.12	-0.04/0.73	0.09/0.48
**ARG**	-0.15/0.22	0.01/0.95	0.07/0.54	0.06/0.59	-0.05/0.70	0.02/0.86	0.22/0.07	0.10/0.40	-0.01/0.96	0.01/0.96	0.00/0.97	0.15/0.19	0.03/0.82	-0.03/0.78	0.14/0.23	0.11/0.36	0.10/0.42	-0.03/0.82	0.04/0.74
**PRO**	-0.22/0.06	0.10/0.40	0.18/0.14	0.16/0.17	0.22/0.06	0.23/0.05	0.10/0.40	0.15/0.21	-0.08/0.52	-0.05/0.68	-0.07/0.57	0.09/0.43	0.23/0.05	0.09/0.44	0.15/0.21	0.12/0.31	0.18/0.14	0.10/0.40	0.26/0.03
**TYR**	-0.18/0.12	0.26/0.02	0.06/0.60	0.14/0.25	0.26/0.03	0.17/0.16	0.13/0.28	0.08/0.49	-0.02/0.86	0.11/0.36	0.06/0.61	0.11/0.35	0.17/0.15	0.35/0.00	0.36/0.00	0.00/0.97	0.15/0.22	0.13/0.28	0.18/0.13
**VAL**	-0.22/0.07	0.15/0.22	0.19/0.11	0.07/0.59	0.22/0.06	0.18/0.13	0.07/0.53	0.01/0.95	-0.12/0.30	0.08/0.53	-0.03/0.81	0.18/0.14	-0.05/0.69	0.28/0.02	0.32/0.01	-0.12/0.33	0.15/0.20	0.08/0.51	0.15/0.22
**MET**	-0.09/0.43	0.11/0.34	0.08/0.52	0.03/0.79	0.12/0.33	0.05/0.65	0.09/0.46	0.00/0.97	-0.12/0.33	-0.01/0.97	0.14/0.25	0.20/0.09	-0.02/0.87	0.25/0.03	0.27/0.02	0.12/0.33	0.10/0.40	0.10/0.39	0.02/0.86
**ILE**	-0.22/0.06	0.15/0.21	0.17/0.14	0.10/0.41	0.17/0.15	0.17/0.15	0.09/0.47	0.00/0.97	-0.11/0.37	0.04/0.72	-0.02/0.87	0.23/0.05	-0.06/0.62	0.26/0.03	0.36/0.00	-0.03/0.81	0.14/0.23	0.07/0.55	0.14/0.25
**LEU**	-0.26/0.03	0.19/0.11	0.16/0.18	0.10/0.38	0.24/0.04	0.14/0.23	0.11/0.38	0.02/0.84	-0.11/0.37	0.06/0.62	0.04/0.72	0.20/0.09	-0.02/0.89	0.32/0.01	0.40/0.00	-0.06/0.63	0.17/0.15	0.09/0.46	0.15/0.22
**ORN**	0.08/0.49	-0.05/0.68	0.06/0.64	-0.03/0.77	0.08/0.52	0.10/0.42	0.05/0.67	-0.11/0.36	-0.04/0.72	-0.05/0.68	-0.03/0.79	0.11/0.37	0.00/0.99	0.05/0.70	-0.09/0.43	0.04/0.73	0.08/0.49	0.26/0.03	0.10/0.39
**PHE**	-0.10/0.39	0.16/0.18	0.20/0.09	0.07/0.55	0.10/0.41	0.11/0.34	0.10/0.39	0.03/0.79	-0.03/0.77	0.09/0.43	0.00/0.99	0.07/0.54	0.04/0.76	0.21/0.08	0.25/0.03	0.04/0.72	0.17/0.16	0.16/0.17	0.15/0.21
**LYS**	-0.12/0.31	0.12/0.33	0.05/0.65	0.00/0.97	0.17/0.16	0.05/0.71	0.06/0.63	0.00/0.97	-0.17/0.15	0.02/0.87	-0.07/0.56	0.17/0.16	-0.08/0.48	0.23/0.05	0.28/0.02	-0.05/0.69	0.09/0.47	0.05/0.66	0.06/0.60
**TRP**	0.19/0.10	-0.06/0.62	0.05/0.69	0.04/0.73	0.13/0.28	0.10/0.38	0.16/0.17	-0.02/0.89	-0.01/0.96	0.18/0.13	0.06/0.61	0.19/0.11	-0.01/0.95	0.06/0.64	0.07/0.58	0.04/0.73	0.05/0.68	0.10/0.39	-0.19/0.11

Supplementary Table 2: Output of spearman's rank sum correlation test for constipation group (FC & IBS-C). Values shown as Rho/significance value.

	PEA	DOPA	DA	X3MT	DOPAC	HVA	NE	DHPG	MHPG	NM	E	MN	VMA	KYN	5.HTP	5.HT	X.HIAA	AABA	GABA
**ASP**	-0.05/0.73	0.06/0.69	0.18/0.24	0.26/0.08	-0.08/0.62	-0.02/0.91	0.04/0.80	-0.22/0.14	-0.21/0.17	0.01/0.96	-0.02/0.88	-0.08/0.62	0.05/0.76	-0.10/0.50	-0.21/0.17	0.34/0.02	0.04/0.80	0.08/0.58	0.08/0.59
**GLU**	0.32/0.03	-0.38/0.01	0.01/0.93	0.05/0.74	-0.11/0.48	-0.02/0.88	-0.09/0.54	-0.30/0.05	-0.45/0.00	0.10/0.53	-0.08/0.60	0.15/0.32	-0.20/0.18	-0.15/0.32	-0.32/0.03	-0.06/0.70	0.02/0.89	-0.14/0.34	-0.29/0.06
**HyPro**	-0.13/0.39	0.10/0.51	0.30/0.05	0.35/0.02	0.09/0.57	0.10/0.50	0.09/0.56	0.24/0.11	0.15/0.34	0.03/0.84	-0.07/0.65	-0.08/0.62	0.23/0.13	-0.05/0.73	0.06/0.70	0.15/0.34	0.25/0.10	0.10/0.52	0.23/0.13

**SER**	0.02/0.91	0.42/0.00	0.12/0.42	0.00/0.99	0.29/0.06	0.32/0.04	0.15/0.31	0.30/0.04	0.26/0.08	-0.10/0.51	0.27/0.08	-0.35/0.02	0.33/0.03	0.27/0.07	0.11/0.48	0.08/0.62	-0.02/0.90	0.24/0.11	0.18/0.23
**ASN**	-0.28/0.06	0.46/0.00	0.26/0.09	0.19/0.21	0.13/0.38	0.32/0.03	0.21/0.16	0.33/0.03	0.37/0.01	0.09/0.56	0.17/0.26	-0.17/0.26	0.46/0.00	0.30/0.05	0.37/0.01	0.15/0.31	0.08/0.58	0.44/0.00	0.61/0.00
**GLY**	0.26/0.08	0.01/0.95	-0.08/0.59	-0.27/0.07	0.29/0.05	-0.05/0.73	-0.05/0.75	0.08/0.59	0.11/0.48	-0.14/0.36	0.28/0.07	-0.23/0.13	0.03/0.82	0.17/0.25	-0.15/0.33	0.01/0.94	-0.12/0.44	-0.06/0.71	-0.03/0.85
**GLN**	0.14/0.37	0.13/0.39	0.10/0.50	0.09/0.54	0.13/0.39	0.28/0.06	0.20/0.18	0.04/0.79	0.09/0.58	0.06/0.70	0.32/0.03	-0.21/0.16	0.19/0.20	0.17/0.27	-0.01/0.96	0.10/0.50	0.03/0.84	-0.05/0.74	0.05/0.72
**HIS**	-0.10/0.53	0.23/0.13	0.21/0.17	0.14/0.35	0.13/0.39	0.32/0.03	0.11/0.47	0.08/0.58	0.22/0.14	0.06/0.70	0.33/0.03	-0.12/0.45	0.29/0.05	0.15/0.32	0.19/0.20	0.21/0.16	0.12/0.43	0.18/0.24	0.33/0.02
**TAU**	-0.16/0.30	0.15/0.32	0.11/0.46	0.21/0.17	-0.15/0.34	-0.16/0.29	0.12/0.44	0.05/0.75	-0.02/0.89	0.01/0.94	0.09/0.58	-0.06/0.69	0.00/0.99	-0.09/0.57	-0.05/0.76	0.46/0.00	-0.07/0.65	0.08/0.58	0.11/0.48
**CIT**	0.30/0.04	-0.09/0.57	0.08/0.59	-0.10/0.53	0.22/0.15	0.17/0.26	-0.15/0.34	-0.01/0.94	0.06/0.69	-0.07/0.65	0.22/0.15	-0.04/0.77	0.16/0.30	0.16/0.29	-0.03/0.82	0.13/0.41	0.06/0.71	0.01/0.96	-0.10/0.51
**THR**	0.00/0.99	0.15/0.34	0.26/0.08	0.21/0.17	0.10/0.49	0.46/0.00	0.14/0.37	0.31/0.04	0.22/0.15	0.20/0.20	0.14/0.37	0.03/0.86	0.40/0.01	0.24/0.11	0.23/0.12	0.10/0.50	0.17/0.27	0.28/0.06	0.38/0.01
**ALA**	0.05/0.74	0.04/0.81	0.23/0.13	0.26/0.08	-0.21/0.17	0.16/0.30	0.14/0.37	0.18/0.25	0.28/0.06	0.23/0.13	0.16/0.30	-0.06/0.69	0.48/0.00	-0.14/0.36	-0.02/0.88	0.24/0.12	0.08/0.60	-0.04/0.81	0.27/0.07
**ARG**	0.17/0.26	0.19/0.21	0.05/0.76	-0.03/0.85	0.44/0.00	0.31/0.04	-0.04/0.79	0.23/0.13	0.09/0.58	-0.23/0.13	0.36/0.01	-0.33/0.03	0.27/0.07	0.16/0.29	-0.12/0.44	0.14/0.34	-0.04/0.79	0.14/0.36	-0.07/0.63
**PRO**	-0.06/0.71	0.04/0.81	0.24/0.11	0.24/0.11	-0.12/0.43	0.26/0.08	0.05/0.75	0.12/0.44	0.01/0.95	0.17/0.26	0.16/0.30	0.08/0.62	0.33/0.03	0.08/0.60	0.20/0.19	0.13/0.38	0.13/0.39	0.07/0.65	0.38/0.01
**TYR**	0.08/0.62	0.33/0.03	0.26/0.08	0.26/0.08	0.14/0.34	0.36/0.01	0.11/0.46	0.08/0.61	0.00/1.00	0.27/0.08	0.30/0.04	-0.10/0.53	0.47/0.00	0.07/0.67	0.09/0.55	-0.09/0.54	0.09/0.56	0.30/0.05	0.19/0.20
**VAL**	0.03/0.86	0.21/0.17	0.33/0.03	0.42/0.00	0.09/0.56	0.22/0.14	0.11/0.47	-0.04/0.78	-0.14/0.38	0.12/0.41	-0.03/0.87	0.04/0.79	0.21/0.17	-0.01/0.96	0.05/0.73	0.14/0.37	0.30/0.05	0.20/0.19	0.16/0.30
**MET**	0.16/0.29	0.19/0.22	0.28/0.06	0.24/0.12	0.17/0.26	0.36/0.02	0.07/0.67	0.14/0.37	0.12/0.42	0.18/0.24	0.22/0.14	-0.12/0.45	0.50/0.00	0.10/0.52	0.10/0.51	0.06/0.71	0.16/0.28	0.30/0.04	0.29/0.06
**ILE**	0.12/0.44	0.18/0.25	0.31/0.04	0.36/0.02	0.10/0.53	0.30/0.05	0.15/0.33	0.07/0.66	-0.03/0.82	0.18/0.24	0.05/0.76	0.06/0.70	0.26/0.08	0.03/0.82	0.08/0.61	0.15/0.34	0.24/0.11	0.15/0.31	0.25/0.10
**LEU**	0.06/0.68	0.23/0.13	0.33/0.03	0.34/0.02	0.14/0.37	0.32/0.03	0.11/0.46	0.03/0.84	-0.08/0.62	0.17/0.25	0.07/0.64	0.04/0.79	0.29/0.06	0.11/0.48	0.14/0.35	0.08/0.62	0.21/0.17	0.23/0.13	0.24/0.11
**ORN**	0.23/0.13	0.06/0.70	0.19/0.21	0.05/0.74	0.16/0.28	0.08/0.58	0.06/0.71	0.03/0.85	0.10/0.50	0.03/0.86	0.33/0.03	-0.07/0.65	0.32/0.03	0.20/0.18	0.11/0.48	-0.01/0.95	0.24/0.12	0.00/1.00	0.20/0.18
**PHE**	-0.02/0.90	0.26/0.08	0.35/0.02	0.27/0.07	0.21/0.17	0.32/0.03	0.11/0.46	0.16/0.30	0.10/0.53	0.19/0.21	0.26/0.09	-0.01/0.95	0.48/0.00	0.13/0.41	0.25/0.09	-0.16/0.29	0.17/0.27	0.31/0.04	0.26/0.08
**LYS**	0.14/0.34	0.24/0.12	0.15/0.32	0.09/0.55	0.29/0.05	0.23/0.14	0.05/0.76	0.15/0.32	0.00/0.99	-0.08/0.59	0.24/0.11	0.01/0.96	0.28/0.07	0.22/0.15	0.07/0.63	0.31/0.04	0.14/0.38	0.14/0.35	0.18/0.24
**TRP**	0.36/0.01	0.03/0.84	0.21/0.16	-0.02/0.89	0.43/0.00	0.29/0.05	0.01/0.93	0.21/0.17	-0.04/0.78	0.04/0.78	0.12/0.45	0.15/0.32	0.13/0.40	0.08/0.58	-0.05/0.72	0.04/0.81	0.02/0.90	-0.01/0.94	-0.21/0.16

Supplementary Table 3: Output of spearman's rank sum correlation test for diarrhea group (FD & IBS-D). Values shown as Rho/significance value.

	**PEA**	**DOPA**	**DA**	**3MT**	**DOPAC**	**HVA**	**NE**	**DHPG**	**MHPG**	**NM**	**E**	**MN**	**VMA**	**KYN**	**5.HTP**	**5.HT**	**5.HIAA**	**AABA**	**GABA**
**ASP**	-0.04/0.76	0.27/0.07	0.04/0.80	0.08/0.58	-0.01/0.97	0.31/0.03	0.21/0.15	0.30/0.04	-0.04/0.79	0.10/0.51	0.26/0.07	-0.24/0.10	0.37/0.01	0.03/0.83	0.06/0.68	0.37/0.01	0.25/0.09	0.08/0.60	0.02/0.87
**GLU**	0.29/0.05	0.14/0.33	-0.17/0.25	-0.08/0.60	-0.01/0.92	0.15/0.30	-0.03/0.84	-0.11/0.46	-0.23/0.12	0.13/0.39	0.20/0.16	-0.02/0.88	0.32/0.03	0.15/0.31	0.01/0.93	0.17/0.24	0.11/0.45	-0.03/0.82	-0.05/0.76
**HyPro**	-0.18/0.22	0.02/0.92	0.08/0.60	0.19/0.20	-0.02/0.91	-0.03/0.82	-0.13/0.38	-0.17/0.25	-0.26/0.07	0.07/0.65	-0.07/0.64	-0.06/0.69	-0.15/0.30	0.07/0.61	0.10/0.49	-0.28/0.06	0.00/0.99	0.23/0.11	0.30/0.04
**SER**	0.00/0.99	0.10/0.50	0.31/0.03	-0.01/0.95	0.24/0.10	0.25/0.08	-0.16/0.29	0.04/0.77	-0.12/0.42	-0.02/0.90	-0.01/0.96	-0.16/0.27	0.23/0.12	0.07/0.64	-0.24/0.10	-0.26/0.08	0.02/0.88	0.23/0.12	0.31/0.03
**ASN**	-0.17/0.25	0.31/0.03	0.23/0.11	-0.06/0.66	0.21/0.15	0.33/0.02	-0.23/0.11	0.13/0.38	-0.18/0.23	-0.27/0.06	-0.08/0.57	-0.36/0.01	0.19/0.20	0.09/0.55	-0.10/0.52	-0.11/0.46	0.21/0.14	0.11/0.47	0.29/0.05
**GLY**	-0.09/0.53	0.01/0.94	0.15/0.30	0.00/0.98	0.21/0.15	-0.03/0.82	-0.04/0.78	0.05/0.74	0.04/0.77	0.13/0.38	0.12/0.40	0.07/0.63	0.17/0.24	-0.05/0.71	-0.28/0.06	-0.16/0.28	-0.03/0.84	0.24/0.11	0.20/0.18

**GLN**	-0.09/0.54	0.30/0.04	0.19/0.20	0.07/0.62	0.12/0.41	0.12/0.42	0.04/0.81	0.30/0.04	0.10/0.49	0.02/0.91	0.21/0.16	-0.31/0.03	0.17/0.24	-0.06/0.71	-0.20/0.18	-0.16/0.27	0.11/0.45	0.24/0.11	0.16/0.27
**HIS**	-0.30/0.04	0.29/0.05	0.07/0.63	-0.10/0.50	0.21/0.15	-0.08/0.61	-0.18/0.23	0.11/0.45	-0.16/0.29	-0.31/0.03	-0.03/0.85	-0.32/0.03	0.09/0.54	0.25/0.08	0.19/0.19	-0.07/0.62	0.20/0.17	0.14/0.34	0.15/0.31
**TAU**	-0.12/0.42	0.21/0.15	0.10/0.50	0.06/0.67	0.05/0.75	0.24/0.10	0.23/0.12	0.31/0.03	0.09/0.56	0.10/0.52	0.30/0.04	-0.22/0.13	0.33/0.02	-0.02/0.91	0.16/0.29	0.36/0.01	0.22/0.14	0.17/0.26	0.03/0.85
**CIT**	0.15/0.32	-0.14/0.33	-0.11/0.45	-0.14/0.36	0.36/0.01	0.19/0.21	0.07/0.65	0.03/0.83	-0.02/0.91	0.14/0.36	0.26/0.07	-0.07/0.62	0.29/0.04	-0.02/0.90	-0.14/0.33	0.04/0.81	0.08/0.58	0.21/0.14	0.03/0.82
**THR**	-0.01/0.94	0.20/0.16	0.13/0.39	-0.07/0.63	0.08/0.60	-0.01/0.92	-0.48/0.00	-0.16/0.26	-0.35/0.02	-0.35/0.01	-0.13/0.38	-0.28/0.05	-0.03/0.85	0.08/0.59	-0.18/0.23	-0.16/0.27	-0.03/0.82	0.10/0.49	0.16/0.27
**ALA**	0.02/0.90	0.59/0.00	0.08/0.59	-0.02/0.89	-0.09/0.54	0.13/0.40	-0.07/0.64	-0.15/0.29	-0.26/0.08	-0.08/0.59	0.04/0.81	-0.27/0.06	0.13/0.39	0.27/0.06	0.03/0.82	-0.11/0.45	0.27/0.06	-0.01/0.96	-0.04/0.77
**ARG**	-0.24/0.11	0.10/0.52	0.24/0.10	0.04/0.79	0.21/0.14	0.19/0.20	-0.09/0.52	0.14/0.35	-0.12/0.41	-0.14/0.33	0.09/0.53	-0.33/0.02	0.05/0.75	0.00/1.00	0.01/0.92	-0.08/0.59	0.07/0.62	0.17/0.24	0.19/0.19
**PRO**	0.03/0.83	0.31/0.03	0.20/0.18	0.14/0.36	-0.01/0.96	0.11/0.45	-0.13/0.37	-0.16/0.28	-0.35/0.02	-0.06/0.66	-0.04/0.77	-0.17/0.24	0.02/0.89	0.05/0.75	-0.02/0.87	-0.09/0.54	0.09/0.55	-0.08/0.58	0.27/0.06
**TYR**	0.02/0.91	0.36/0.01	0.07/0.64	-0.14/0.33	0.02/0.90	0.31/0.03	-0.13/0.36	0.00/0.99	-0.08/0.61	-0.02/0.89	-0.05/0.74	-0.32/0.03	0.26/0.08	0.13/0.39	0.06/0.69	0.08/0.59	0.11/0.47	-0.12/0.43	0.29/0.05
**VAL**	-0.04/0.80	0.34/0.02	0.10/0.52	0.03/0.82	-0.07/0.61	0.25/0.08	-0.10/0.48	-0.04/0.77	-0.23/0.11	0.04/0.77	0.02/0.88	-0.11/0.46	0.12/0.43	0.17/0.25	0.12/0.43	-0.11/0.46	0.10/0.51	-0.18/0.23	0.13/0.36
**MET**	-0.04/0.79	0.47/0.00	0.10/0.50	-0.04/0.78	0.26/0.08	0.30/0.04	-0.10/0.49	0.11/0.47	-0.08/0.58	-0.03/0.83	0.09/0.53	-0.20/0.16	0.32/0.03	0.37/0.01	0.08/0.61	0.13/0.37	0.24/0.10	-0.17/0.23	0.13/0.37
**ILE**	-0.06/0.68	0.33/0.02	0.08/0.60	0.04/0.79	-0.01/0.95	0.28/0.05	-0.11/0.46	0.03/0.86	-0.21/0.14	0.05/0.74	0.12/0.43	-0.03/0.83	0.13/0.38	0.27/0.07	0.23/0.12	0.01/0.92	0.04/0.77	-0.15/0.29	0.22/0.14
**LEU**	-0.16/0.27	0.32/0.03	0.07/0.63	0.02/0.89	0.06/0.68	0.36/0.01	-0.10/0.51	0.08/0.59	-0.11/0.45	0.05/0.71	-0.01/0.96	-0.07/0.66	0.21/0.15	0.19/0.21	0.19/0.19	0.06/0.67	0.11/0.45	-0.13/0.38	0.28/0.05
**ORN**	0.27/0.06	0.19/0.20	0.23/0.12	0.05/0.74	0.10/0.48	0.10/0.48	-0.15/0.31	-0.21/0.15	-0.37/0.01	0.20/0.18	-0.03/0.87	-0.14/0.34	0.12/0.44	0.19/0.19	-0.01/0.95	-0.04/0.77	0.13/0.38	0.11/0.47	0.24/0.11
**PHE**	0.06/0.68	0.41/0.00	0.09/0.56	-0.08/0.58	0.02/0.90	0.25/0.09	0.17/0.25	0.26/0.07	0.05/0.73	0.15/0.31	0.23/0.12	-0.16/0.28	0.41/0.00	0.36/0.01	0.24/0.10	0.13/0.38	0.07/0.63	-0.05/0.74	0.01/0.94
**LYS**	-0.09/0.56	0.22/0.14	-0.03/0.83	-0.16/0.29	0.26/0.08	0.04/0.79	-0.23/0.12	-0.15/0.30	-0.14/0.34	-0.17/0.25	0.02/0.89	-0.26/0.08	0.10/0.48	0.37/0.01	0.27/0.07	-0.18/0.23	0.05/0.73	0.09/0.53	0.08/0.58
**TRP**	0.49/0.00	0.16/0.29	0.15/0.29	-0.16/0.28	0.10/0.51	0.03/0.84	0.04/0.76	-0.03/0.87	-0.04/0.77	-0.04/0.79	-0.04/0.77	-0.08/0.59	0.14/0.33	0.05/0.72	-0.08/0.60	0.09/0.53	0.00/1.00	-0.07/0.65	-0.37/0.01
